# Factors Associated with Blood Pressure Control in Hypertensive Patients with Coronary Heart Disease: Evidence from the Chinese Cholesterol Education Program

**DOI:** 10.1371/journal.pone.0063135

**Published:** 2013-05-15

**Authors:** Dachun Xu, Wei Chen, Xiankai Li, Yi Zhang, Xin Li, Hou Lei, Yidong Wei, Weiming Li, Dayi Hu, Nicole M. Wedick, Jinsong Wang, Yawei Xu, Jue Li, Yunsheng Ma

**Affiliations:** 1 Department of Cardiology, Shanghai Tenth People's Hospital, Tongji University School of Medicine, Shanghai, China; 2 Heart, Lung and Blood Vessel Center, Tongji University School of Medicine, Shanghai, China; 3 Department of Cardiology, Shanghai East Hospital, Tongji University School of Medicine, Shanghai, China; 4 Key Laboratory of Arrhythmias of Ministry of Education of China, Tongji University, Shanghai, China; 5 Division of Preventive and Behavioral Medicine, Department of Medicine, University of Massachusetts Medical School, Worcester, Massachusetts, United States of America; 6 Department of Preventive Medicine, Yangzhou University School of Medicine, Yangzhou, China; Universidade Federal do Rio de Janeiro, Brazil

## Abstract

Blood pressure (BP) remains poorly controlled among hypertensive patients with coronary heart disease (CHD) in China. Improvement of its management will require an understanding of the patient characteristics and treatment factors associated with uncontrolled hypertension. A cross-sectional survey of 3,279 patients from 52 centers in China was performed to examine potential barriers to adequate blood pressure control of hypertensive patients with CHD. Uncontrolled hypertension was defined as blood pressure ≥130/or 80 mmHg. Multivariable logistic regression was used to identify factors associated with poor blood pressure control. Mean age of the patients was 65 years, 40% were women, and mean BMI was 25 kg/m^2^. Mean systolic blood pressure was 136±18 mmHg and mean diastolic blood pressure was 80±11 mmHg. Only 18% of patients had a mean blood pressure <130/80 mmHg during the study period. Multivariate analysis revealed several independent factors of poor blood pressure control: body mass index ≥23 kg/m^2^, the presence of stable angina pectoris (SAP), family history of diabetes, and use of calcium channel blockers (CCB). Further analysis showed that non-dihydropyridine calcium antagonist was significantly correlated with low BP control rate. Some of these may be amenable to modification. The results of our study suggest that overweight, the presence of SAP and family history of diabetes are important factors for tight BP control in primary care. In addition, non-dihydropyridine calcium channel blockers appear less effective than other therapies in control of blood pressure and should not be the first choice among hypertensive patients with CHD. Further identification of patients at risk of poor BP control can lead to targeted interventions to improve management.

## Introduction

Hypertension is an important risk factor for cardiovascular disease and has become a major global burden on public health [1]. In 2002, one-sixth of all Chinese adults were found to be hypertensive, and in most cases, hypertension was uncontrolled [2]. In addition, hypertension has often clustered with coronary heart disease (CHD). For example, 46% of hypertensive patients had a history of CHD in the Valsartan Antihypertensive Long-term Use Evaluation (VALUE) trial [3]. Moreover, hypertensive patients with CHD often had uncontrolled blood pressure (BP) as well as higher cardiovascular morbidity and mortality. Uncontrolled blood pressure could augment cardiovascular risk in hypertensive individuals with CHD, so it is important to identify the factors that influence blood pressure control in hypertensive patients with CHD.

Generally, most guidelines stipulated a blood pressure treatment goal of 140/90 mmHg. However, recent clinical trials showed further benefit could be achieved by more aggressive blood pressure lowering to well below 130/80 mmHg in hypertensive populations, including those patients with CHD [4], [5]. Based on these recent trials, American Heart Association Scientific Statement recommended that this lower blood pressure treatment goal be expanded to include patients with CHD, stable or unstable angina pectoris, and myocardial infarction (MI) with or without ST elevation in 2007 [5]. A blood pressure goal of <130/80 mmHg is strongly recommended for hypertensive patients with CHD.

Previous trials did not measure blood pressure control rates among hypertensive patients with CHD. It will therefore be important to identify factors that could affect blood pressure control in hypertensive patients with CHD in a large, nationally representative sample population. The purpose of this study was to use data from a previously reported China Cholesterol Education Program (CCEP) [6] to investigate factors that may influence blood pressure control in hypertensive patients with CHD in China.

## Methods

### Material and Data Collection

The China Cholesterol Education Program (CCEP) was a cross-sectional, large multicenter investigation, which involved 52 centers in 6 cities (Shanghai, Beijing, Guangzhou, Zhejiang, Tianjin and Xinjiang) in China [6]. Participants (n = 4778) were continuously enrolled from January 2006 to January 2007 to investigate blood lipid levels and the prevalence of achieving the goal of low-density lipoprotein-cholesterol (LDL-C) level (<2.6 mmol/L) in Chinese outpatients with CHD. From the CCEP study, 3279 participants were included for investigation of blood pressure control and factors that may influence blood pressure control in hypertensive patients with CHD. Data were collected by questionnaire including demographic data, medical history, family history, CHD diagnosis, treatment of CHD and laboratory examinations. Body mass index (BMI), blood pressure, fasting plasma glucose, lipids, and smoking status were also recorded. Blood pressure measurements were taken in the study clinic by study personnel using a Dinamap XL automated BP monitor. In Asian population, a BMI cutoff of 23 kg/m^2^ was recommended to use to define overweight [7], [8]. We, therefore, use this cut point in our analyses. All participants signed written informed consent statements allowing access to their medical records. The data collection protocols were approved by the Peking University Research Ethics Committee.

### Diagnosis of CHD and comorbidities

CHD was diagnosed physicians, and supported by at least one of the following objective findings; abnormal stress tests (i.e., treadmill electrocardiography, cardiac scintigraphy, or stress echocardiography) indicating significant myocardial ischemia, a coronary angiogram revealing >50% stenosis of the lumen of any major coronary artery, a history of confirmed myocardial infarction (MI) or evidence of prior MI on electrocardiogram, or a history of a prior coronary revascularization procedure (percutaneous coronary intervention or coronary artery bypass graft). The CHD diagnosis was classified as stable angina pectoris (SAP), previous MI, or acute coronary syndromes (ACS), which included unstable angina pectoris, ST-segment elevation MI and non-ST-segment elevation MI [6].

Hypertension was defined as systolic blood pressure ≥140 mmHg, and/or diastolic blood pressure ≥90 mmHg, and/or current antihypertensive medication [9]. Uncontrolled hypertension was defined as blood pressure ≥130/or 80 mmHg. Diabetes was defined as: (1) fasting plasma glucose concentration ≥7.1 mmol/L in the absence of treatment; (2) glucose concentration ≥11.1 mmol/L, 2 h after a 75 g oral glucose load; or (3) current treatment with hypoglycemic drugs [10]. Peripheral artery disease was defined as partial or complete atherosclerotic obstruction of the peripheral arteries on angiography, or intermittent claudication [11]. Dyslipidemia was defined as total cholesterol (TC) ≥5.2 mmol/L, and/or high-density lipoprotein-cholesterol (HDL-C) ≤0.90 mmol/L, and/or LDL-C ≥3.12 mmol/L, and/or triglyceride (TG) ≥1.69 mmol/L, or undergoing current lipid-lowering treatment [6]. Smoking history was considered positive in those who smoked at least 1 cigarette/day for at least 1 year [6].

### Statistical Analysis

All case record form data were entered into dual Epidata 3.02 databases. The databases were compared and necessary corrections were made. All analyses were performed with the Statistics Package for Social Science, version 13.0 (Chicago, IL, USA). Continuous variables are expressed as mean ± SD, and discrete variables as percentages. The differences in continuous variables between groups were examined by t-test. The differences in discrete variables between groups were calculated by the Pearson χ^2^ test. Stepwise logistic regression models were used to estimate the odds ratios (ORs) and associated 95% CI of blood pressure control, and the probability for variable entry into models was 0.05 and for variable removal was 0.10. The variables that were significant (P<0.1) in the bivariate analysis were included in a multivariate logistic regression model. Full model included BMI≥23 kg/m^2^, stable angina pectoris (SAP), family history of diabetes, and CCB.

## Results

**Figure 1:**
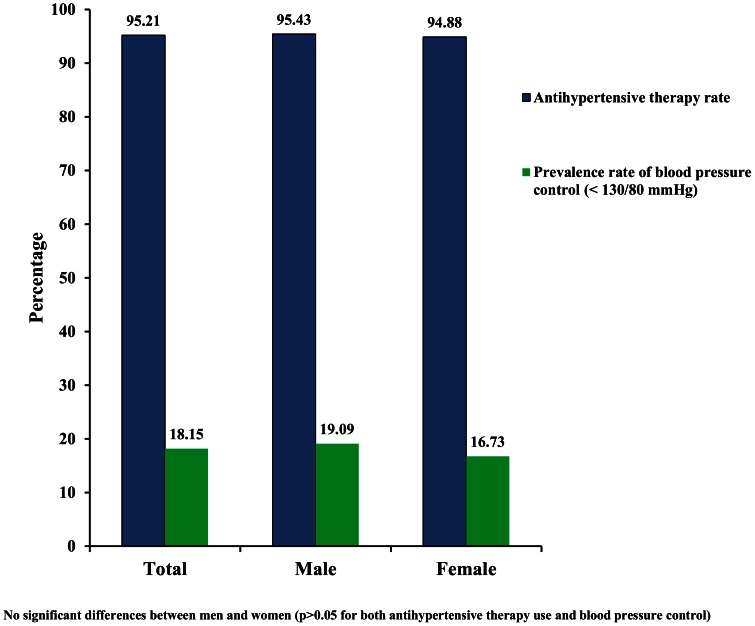
Antihypertensive therapy rate and prevalence of blood pressure control.

This was a multicenter observational study conducted in the Chinese society of cardiology. Demographic and clinical characteristics of hypertensive patients with CHD are listed in Table 1. Among 3279 subjects, the mean age was 65 years, 40% were women, and mean BMI was 25 kg/m^2^ (calculated as weight in kilograms divided by height in meters squared). Table 2 lists all of treatments and concomitant medication for these patients. The most common medication use is statin (83% of participants), followed by β-blockers (64%), CCB (49%), ACEI (46%), ARB (22%), and diuretics (16%). Figure 1 illustrates that nearly 95% of individuals were receiving antihypertensive medication, only 18% individuals achieved tight blood pressure control, and there were no significant differences of percentages of antihypertensive therapy use and blood pressure control between men and women (p>0.05 for both).

**Table 1:** Demographic and clinical characteristics hypertensive patients with CHD, CCEP 2006 (N = 3279).

Parameter	
Categorical variable	n (%)
Female	1309 (40.0%)
Smoking, n (%)	1167 (35.6)
Diabetes mellitus, n (%)	831 (25.3)
Hyperlipidemia, n (%)	1938 (59.1)
Stable angina pectoris, n (%)	1109 (33.8)
Myocardial infarction, n (%)	430 (13.1)
Peripheric atherosclerosis, n (%)	485 (14.8)
Family history of Hypertension, n (%)	1376 (42.0)
Family history of diabetes, n (%)	357 (10.9)
Family history of MI, n (%)	366 (11.2)
Family history of CHD, n (%)	671 (20.5)

Continuous variable	mean ±standard deviation
Age (years)	65.81±10.78
BMI (kg/m^2^)	24.79±3.13
SBP (mmHg)	136.11±17.98
DBP (mmHg)	80.36±10.99
TC (mmol/L)	5.03±1.51
TG (mmol/L)	2.00±1.38
HDL-C (mmol/L)	1.24±0.43
LDL-C (mmol/L)	2.97±1.01
FPG (mmol/L)	6.08±2.79

CCEP: China Cholesterol Education Program; BMI: body mass index; MI: myocardial infarction; CHD: coronary heart disease; SBP: systolic blood pressure; DBP: diastolic blood pressure; TC: cholesterol; TG: triglyceride; HDL-C: high-density lipoprotein cholesterol; LDL-C: low-density lipoprotein cholesterol; FPG: fasting plasma glucose.

**Table 2:** Medication use among hypertensive patients with CHD (N = 3279).

Medication use	n (%)
Diuretics	534 (16.3)
β-blockers	2089 (63.7)
CCB	1597 (48.7)
ACEI	1506 (45.9)
ARB	829 (25.3)
Statins	2698 (82.3)
CCB+Diuretics	243 (7.4)
CCB+β-blockers	974 (29.7)
CCB+ACEI	606 (18.5)
CCB+ARB	426 (13.0)
CCB+Statins	1327 (40.5)
ACEI+Diuretics	259 (7.9)
ACEI+β-blockers	1036 (31.6)
ACEI+ARB	92 (2.8)
ACEI+Statins	1311 (40.0)
Statins+Diuretics	442 (13.5)
Statins+β-blocker	1801 (54.9)
Statins+ARB	680 (20.7)
Diuretics+β-blockers	327 (10.0)
Diuretics+ARB	202 (6.2)
β-blockers+ARB	504 (15.4)

CHD: coronary heart disease; CCB: calcium channel blockers; ACEI: angiotension converting enzyme inhibitor; ARB: angiotension receptor blocker.

**Figure 2:**
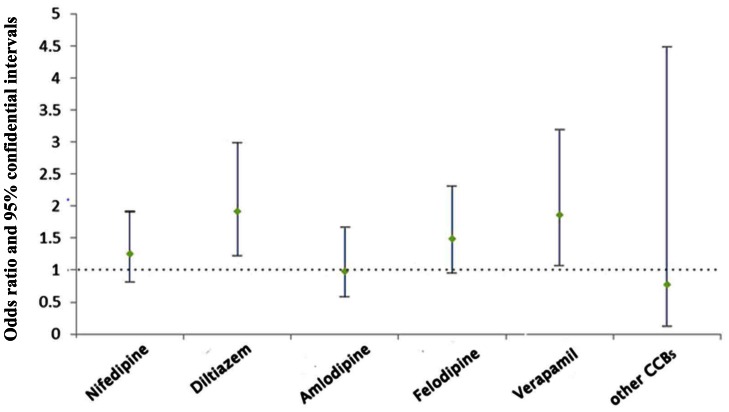
Odds ratios and 95% confidence intervals predicting blood pressure control attainment by type of antihypertensive medication use.

The results of bivariate analysis of factors affecting BP control are shown in Table 3. For hypertensive patients on admission, BP was optimized according to the guidelines with antihypertensive drugs (CCB: calcium channel blockers; ACEI: angiotension converting enzyme inhibitor; ARB: angiotension receptor blocker; diuretics; β-blockers) to attain normotensive BP values. Multivariate logistic regression analysis showed that the following four factors were significantly associated with uncontrolled blood pressure control: higher BMI [OR, 1.426; 95% CI, 1.176–1.729; P<0.001], the presence of SAP (OR, 1.230; 95% CI, 1.013–1.492; P<0.036), family history of diabetes (OR, 1.428; 95% CI, 1.035–1.971; P = 0.030), and use of CCB (OR, 1.360; 95% CI, 1.135–1.629; P = 0.001) (Table 4). Furthermore, multivariate logistic regression analysis showed that non-dihydropyridine calcium antagonist was significantly correlated with low BP control (Figure 2).

**Table 3:** Bivariate analysis of factors affecting blood pressure control in hypertensive patients with CHD.

	Controlled (n = 595)	Uncontrolled (n = 2684)	*P* –value
**Characteristics**			
Age (years)	66.57±10.60	65.64±10.81	0.059
Female, n (%)	219 (36.8)	1090 (40.6)	0.086
BMI≥23 kg/m^2^, n (%)	395 (66.4)	1990 (74.1)	<0.001
Smoking, n (%)	216 (36.3)	951 (35.4)	0.688
**Diseases history**			
Diabetes mellitus, n (%)	162 (27.2)	669 (24.9)	0.221
Myocardial infarction, n (%)	80 (13.5)	350 (13.0)	0.755
Peripheric atherosclerosis, n (%)	88 (14.8)	397 (14.8)	0.999
Hyperlipidemia, n (%)	339 (57.0)	1599 (59.6)	0.309
Stable angina pectoris, n (%)	180 (30.3)	929 (34.6)	0.042
Family history of hypertension, n (%)	240 (40.3)	1136 (42.3)	0.374
Family history of diabetes, n (%)	48 (8.1)	309 (11.5)	0.015
Family history of MI, n (%)	60 (10.1)	306 (11.4)	0.356
Family history of CHD, n (%)	114 (19.2)	557 (20.8)	0.384
**Medication use**			
Diuretics, n (%)	101 (17.0)	433 (16.1)	0.615
β-blocker, n (%)	380 (63.9)	1709 (63.7)	0.930
CCB, n (%)	250 (42.0)	1347 (50.2)	<0.001
ACEI, n (%)	276 (46.4)	1230 (45.8)	0.804
ARB, n (%)	167 (28.1)	662 (24.7)	0.084
Statins, n (%)	507 (85.2)	2191 (81.6)	0.050
CCB+Diuretics, n (%)	34 (5.7)	209 (7.8)	0.081
CCB+β-blocker, n (%)	156 (26.2)	818 (30.5)	0.040
CCB+ACEI, n (%)	93 (15.6)	513 (19.1)	0.048
CCB+ARB, n (%)	68 (11.4)	358 (13.3)	0.210
ACEI+Diuretics, n (%)	41 (6.9)	218 (8.1)	0.314
ACEI+β-blocker, n (%)	193 (32.4)	843 (31.4)	0.625
ACEI+ARB, n (%)	21 (3.5)	71 (2.7)	0.237
Diuretics+β-blocker, n (%)	62 (10.4)	265 (9.9)	0.687
Diuretics+ARB, n (%)	44 (7.4)	158 (5.9)	0.166
β-blocker+ARB, n (%)	100 (16.8)	404 (15.1)	0.283

CHD: coronary heart disease; BMI: body mass index; MI: myocardial infarction; CCB: calcium channel blockers; ACEI: angiotension converting enzyme inhibitor; ARB: angiotension receptor blocker.

**Table 4:** Final multivariate model predicting blood pressure control in hypertensive patients with CHD.

Variable	*OR*	*95% CIs* Lower limit	*95% CIs* Upper limit	*P* -value
BMI≥23 kg/m^2^	1.426	1.176	1.729	<0.001
Stable angina pectoris	1.230	1.013	1.492	0.036
Family history of diabetes	1.428	1.035	1.971	0.030
CCB	1.360	1.135	1.629	0.001

CHD: coronary heart disease; BMI: body mass index; CCB: calcium channel blockers; OR: odds ratios; 95% CIs: 95% confidential intervals.

## Discussion

The study represents the first analysis of factors that may be associated with blood pressure control in hypertensive patients with CHD. We observed that nearly 95% of individuals were receiving antihypertensive medication, but only 16.7% subjects (female) and 19.1% (male) had adequate BP control (<130/80 mmHg). Results from multivariate logistic regression analysis show that overweight, the presence of SAP, and family history of diabetes contribute to low BP control rate in these populations. Further analysis of the data document that non-dihydropyridine calcium antagonist are also significantly correlated with low BP control.

Previous meta-regression analysis demonstrated that the benefit of antihypertensive drug treatment was largely attributable to BP reduction [12]. Blood Pressure Lowering Treatment Trialists' Collaboration (BPLTTC) reported that the reduction in CHD and stroke produced by antihypertensive treatment increased with low BP targets in studies comparing tight to usual BP control [13], and other clinical trials have also provided evidence of reduced incidence of cardiovascular events by bringing blood pressure to rather low levels in patients with angina pectoris or coronary heart disease: the EUropean trial on Reduction Of cardiac events with Perindopril in patients with stable coronary Artery disease (EUROPA) study: 128/78 rather than 133/80 mm Hg [4]; the Comparison of Amlodipine vs Enalapril to Limit Occurrences of Thrombosis (CAMELOT) study: 124/76 rather than 130/77 mmHg [14]). The European Society of Cardiology (ESC) 2007 guideline recommended that target BP should be at least less than 130/80 mm Hg in diabetics and in high or very high risk patients. Data from patients with coronary artery bypass surgery of the global REduction in Atherothrombosis for Continued Health (REACH) Registry showed that 76.3% reached BP control [15].However, only 16.7% subjects (female) and 19.1% (male) had adequate BP control (<130/80 mmHg) in our patient population. Therefore, it is important to identify factors that influence controllability of blood pressure.

In our present study, we found a significant association of uncontrolled BP with the presence of obesity, SAP and family history of DM, which were consistent with other previous publications [16], [17]. Cardiac autonomic dysfunction may explain poor blood pressure control in overweight and obese hypertensive patients with CHD [16].Actually, these metabolism-related disorders, mainly with elevated plasma glucose and cholesterol, would have a considerable impact not only on the atherosclerosis, such as in patients with CHD, but also on arteriolosclerosis, a condition with increased arterial stiffness. Recently, in the Framingham Heart study, arterial stiffness was considered to be an important and indispensable cause of new-onset hypertension, but hypertension would not lead to the increased arterial stiffness [17].Together these studies, our findings indicated that treatment on either those metabolism-related disorders or arterial stiffness, would eventually contribute to improved BP control.

Uncontrolled high blood pressure can lead to a variety of changes in the myocardial structure, coronary vasculature, and conduction system of the heart. These changes in turn can lead to the development of left ventricular hypertrophy, coronary artery disease, and systolic and diastolic dysfunction of the myocardium, complications that manifest clinically as angina [18]. The role of family history of DM on uncontrolled BP may be due to the dietary habit [19]. As we know there is significant association between poor diet and risk of diabetes, poor diet tends to have high sodium intake, which was associated poor BP control [20].

Multivariate logistic regression analysis showed poor control of blood pressure was also associated with use of calcium channel blockers (CCB). Although several studies have shown improvements in the outcomes of patients with CHD using the dihydropyridine calcium channel blocker [21], [22], there have been few reports of such vascular protection effects of calcium channel blockers other than amlodipine. Dihydropyridines tend to be more potent vasodilators than non-dihydropyridine agents (diltiazem, verapamil), whereas the latter have marked negative inotropic effects. Non-dihyrdropyridine calcium channel blockers were taken more often by uncontrolled than controlled hypertensive patients [16].Among patients receiving only one drug, non-dihydropyridine calcium channel blockers are associated with less nocturnal BP decline than other antihypertensive drug classes, even after adjusting for the level of risk [23]. These results are consistent with our study. However, CCB are substances with potential antianginous properties, used for CHD treatment. Even if the antihypertensive potential is rather low, it might be a useful tool in treatment of SAP, certainly not as a first line antihypertensive medication [24].

The present analysis has several potential limitations. First, the analysis is cross-sectional, and we cannot determine temporal direction or causality. Second, because the present analysis is performed from an aspect differing from the original purpose of observation, a bias in the selection of patients cannot be excluded. Another limitation is potential measurement error from the medication use data. We do not have information on actual adherence to the treatment regimen. In addition, because the dosages of CCB including non-dihydropyridine calcium channel blockers and their continuation states are not investigated, the effective dosages of CCB could not be clarified. Further studies are necessary to clarify these points. However, these data have relevant clinical implications, suggesting that physicians pay close attention toward the rational use of CCB. The current analysis also extends our understanding of cross-sectional covariates associated with hypertensive patients with CHD.

Our data suggest that the presence of obesity, SAP and family history of diabetes are important factors for tight BP control in adults with CHD. In addition, non-dihydropyridine calcium channel blockers may be less effective than other therapies in control of blood pressure and should not be the first choice among hypertensive patients with CHD.
